# Advancing Tumor Therapy: Development and Utilization of Protein-Based Nanoparticles

**DOI:** 10.3390/pharmaceutics16070887

**Published:** 2024-07-01

**Authors:** Shirin Khakpour, Nushin Hosano, Zahra Moosavi-Nejad, Amir A. Farajian, Hamid Hosano

**Affiliations:** 1 Graduate School of Science and Technology, Kumamoto University, Kumamoto 860-8555, Japan; 230d9364@st.kumamoto-u.ac.jp; 2 Department of Biomaterials and Bioelectrics, Institute of Industrial Nanomaterials, Kumamoto University, Kumamoto 860-8555, Japan; nushin@kumamoto-u.ac.jp; 3 Department of Biotechnology, Faculty of Biological Sciences, Alzahra University, Tehran 1993893973, Iran; 4 Department of Mechanical and Materials Engineering, Wright State University, Dayton, OH 45435, USA; amir.farajian@wright.edu

**Keywords:** protein nanoparticles (PNPs), drug delivery, nanoparticle synthesis techniques, biomedicine, tumor therapy

## Abstract

Protein-based nanoparticles (PNPs) in tumor therapy hold immense potential, combining targeted delivery, minimal toxicity, and customizable properties, thus paving the way for innovative approaches to cancer treatment. Understanding the various methods available for their production is crucial for researchers and scientists aiming to harness these nanoparticles for diverse applications, including tumor therapy, drug delivery, imaging, and tissue engineering. This review delves into the existing techniques for producing PNPs and PNP/drug complexes, while also exploring alternative novel approaches. The methods outlined in this study were divided into three key categories based on their shared procedural steps: solubility change, solvent substitution, and thin flow methods. This classification simplifies the understanding of the underlying mechanisms by offering a clear framework, providing several advantages over other categorizations. The review discusses the principles underlying each method, highlighting the factors influencing the nanoparticle size, morphology, stability, and functionality. It also addresses the challenges and considerations associated with each method, including the scalability, reproducibility, and biocompatibility. Future perspectives and emerging trends in PNPs’ production are discussed, emphasizing the potential for innovative strategies to overcome current limitations, which will propel the field forward for biomedical and therapeutic applications.

## 1. Introduction

Particle technology, encompassing the science and technology of particles and powders, is crucial across various industries such as chemicals, food, pharmaceuticals, and metals [1]. Notably, there has been a significant transition from macro to micro and nano scales, with Richard Feynman introducing the concept of nanotechnology in 1959 [2]. Subsequently, Norio Taniguchi defined nanotechnology in 1974 as the manipulation of materials at the atomic or molecular levels [3]. Nanomaterials, fundamental in nanoscience and nanotechnology, exhibit unique properties and were defined by the International Organization for Standardization (ISO) as substances within the nanoscale range. However, the precise definitions remain debated [4,5,6]. Nanomaterials possess distinctive characteristics depending on their size, structural arrangement, origin, pore size, and probable toxicity, highlighting their versatility, leading to the classification of these materials into different categories [4,5,7,8].

Nanomaterials can be broadly categorized based on their composition into carbon-based nanomaterials, metallic nanomaterials, ceramic-based nanomaterials, polymeric nanomaterials, and nanomaterials derived from biomolecules. Carbon-based nanomaterials, like carbon nanotubes (CNTs), graphene, and carbon quantum dots (CQDs), are widely used due to their exceptional properties. They find applications in diverse fields such as water treatment, electronics, biomedicine, and polymers [5,9,10]. Metallic nanoparticles, including gold (Au) and silver (Ag) nanoparticles, exhibit remarkable properties compared to their bulk counterparts, enabling applications in medicine, electronics, energy, and catalysis [11,12,13,14,15]. Ceramic nanomaterials, like silica and alumina-based ones, are valued for their heat resistance and chemical inertness, finding applications in biomedicine despite the concerns about biodegradability and toxicity [4,5,9,16]. Polymeric nanomaterials, including natural and biosynthesis-based ones, are notable for drug delivery, separation, and surface coatings due to their biodegradability and safety [17,18,19]. Biomolecule-derived nanomaterials offer potential in drug delivery and biosensing, either alone or in hybrid forms with inorganic nanomaterials [20,21,22,23]. Among nanomaterials, nanoparticles are defined by the ISO as particles with one or more dimensions of the order of 100 nm or less [24].

The recent progress has been driven by integrating nanotechnology with biotechnology, resulting in novel biological nanoparticles and biomolecule–nanoparticle conjugates with diverse applications. In particular, protein nanoparticles (PNPs) have garnered significant attention in recent years for their potential applications in various fields, including drug delivery, biomedical imaging, and tissue engineering. These nanoparticles, derived from proteins or peptides, offer unique advantages such as biocompatibility, which is the capacity of a biomaterial to execute its intended purpose without causing any harmful or toxic reactions within biological systems, while simultaneously evoking an appropriate response from the host in a particular case in clinical use [25]. They also offer tunable properties and versatile functionality, making them attractive platforms for innovative therapeutic strategies. The precise control over nanoparticle size, morphology, and surface characteristics allows tailored designs to meet specific biomedical needs [26,27]. PNPs exhibit considerable potential in the field of targeted therapies, including cancer treatment and vaccines. Utilizing PNPs as nanocarriers for anticancer drugs has proven effective in augmenting their antitumor potency while simultaneously mitigating the toxicities linked to the traditional formulations of such agents. The production of PNPs and PNP/drug conjugates encompasses a range of methodologies, each with its own principles, advantages, and challenges, which can affect the final therapeutic outcomes.

This review aims to examine in depth the existing techniques employed in PNPs’ production. In order to develop effective PNPs and PNP/drug complexes for tumor therapy, it is necessary to understand the nanoparticle production methods, so that nanoparticle properties can be optimized to enhance drug delivery and minimize the off-target effects, ultimately resulting in an appropriate outcome for cancer patients. The PNP synthesis methods in the current study have been classified into three primary categories: solubility change, solvent substitution, and thin flow methods. This categorization provides a concise framework for organizing the various techniques employed in PNP synthesis. Furthermore, these categories demonstrate a direct link to the behavior of proteins across different scenarios. This further emphasizes the importance of factors such as solubility, solvent interactions, and fluid dynamics in the formation of nanoparticles. Additionally, the recent advancements in protein engineering and bioconjugation techniques have further expanded the possibilities for designing PNPs with enhanced functionalities and targeted delivery capabilities. By consolidating the current knowledge and addressing the emerging trends, this review explores how the fabrication methods can affect PNPs’ therapeutic applications.

## 2. Nanomaterials in Biomedical Fields

Nanotechnology holds immense promise in biomedicine, offering innovative solutions for diagnosis and treatment [28,29,30]. Over the recent decades, nanotechnology has made significant strides in tumor therapy, drug delivery, molecular imaging, and diagnostics, leading to the availability of various nano pharmaceuticals in the market [8,30,31,32]. Nanoparticles, with their high surface area to volume ratio, efficiently absorb medications and navigate the bloodstream swiftly, offering unique mechanical, magnetic, optical, and catalytic properties that enhance their pharmaceutical utility [33].

**Figure 1:**
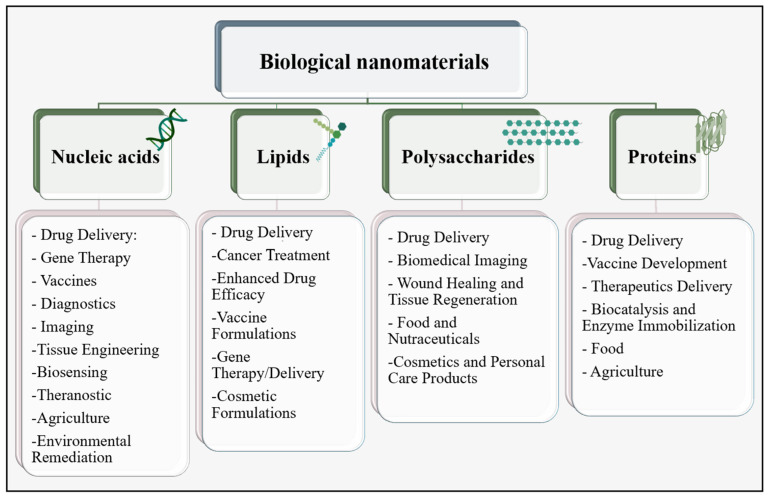
Classification scheme depicting the taxonomy of biological nanoparticles based on their composition. Applications of each category are outlined to provide additional details.

In biomedical applications, nanomaterials fall into three categories based on their composition: organic, inorganic, and carbon-based [33,34]. Organic nanomaterials, such as liposomes and polymersomes, are gaining attention for their structural integrity and controlled release capabilities, while inorganic nanoparticles, including elemental metals and metal oxides, including elemental metals and metal oxides such as those derived from Au or Ag, serve as contrast agents and exhibit stability [33,34,35,36]. Over the past decades, there has been a significant surge in the exploration of biological nanomaterials, which are natural and organic nanomaterials obtained from biomolecules, as a viable substitute for chemically synthesized nanomaterials [7,37]. Biological nanoparticles refer to particles that have a size ranging from 10 nm to 1 μm and are derived from biomolecules or organic compounds. These particles can be classified into four main categories, namely proteins, nucleic acids, lipids, and polysaccharides, based on their composition [38], as shown in Figure 1.

Nucleic acid-based self-assembling nanocarriers (NASNs) have become integral in biomedical research, particularly in drug delivery, release, and targeting [39,40,41]. Recent advancements in nucleic acid nanotechnology have led to the development of nano-assemblies characterized by programmable design, impressive functionality, biocompatibility, and biosafety [39,42]. These structures, constructed from DNA, RNA, or composite materials, exhibit remarkable capabilities in drug transport across physiological barriers, enabling targeted delivery and enhancing drug efficacy while minimizing toxicity.

Lipid-based nanoparticles (LBNPs), such as liposomes, solid lipid nanoparticles (SLNs), and nanostructured lipid carriers (NLCs), are gaining attraction in drug discovery and cancer treatment due to their versatility in transporting both hydrophobic and hydrophilic molecules, low toxicity profile, and ability to improve drug efficacy via prolonged half-life and controlled release mechanisms [43,44,45].

Polysaccharides, natural biopolymers composed of multiple monosaccharide units linked by glycosidic bonds, are valued for their eco-friendly nature [46,47]. Recently, polysaccharide nanoparticles have found wide application across various fields, notably in disease management and therapeutic delivery, offering effective solutions with significant societal impact. Nanocellulose, chitin/chitosan, and starch nanoparticles stand out for biomedical applications due to their biocompatibility, biodegradability, low cytotoxicity, and ample surface functional groups amenable to chemical modification [48,49]. It has been shown that polysaccharide-based nanoparticles can be used in cancer immunotherapy and initiate the desired immune responses which can be interpreted as a good compatibility of these nanoparticles [50]. However, this immune system activation should be considered when immune responses are not desired.

Proteins, with their distinct functionalities and well-defined primary structure, are valuable precursors for creating nanoparticles [11,37]. They offer diverse opportunities for surface modifications and the attachment of substances like drugs, while their versatility allows their processing into gels, emulsions, or dried particles, with enhanced stability in vivo and during storage.

PNPs have demonstrated significant promise in targeted therapies, including pulmonary delivery, cancer treatment, and vaccines. These nanoparticles can be seamlessly integrated into biodegradable polymers as microspheres, facilitating controlled and sustained drug release. PNPs offer several advantages, including biodegradability, low toxicity, and minimal antigenicity [51]. The primary goal in designing nanoparticle-based drug delivery systems is to regulate particle size, surface area, and surface properties to ensure efficient drug carriage and the desired pharmacological activity. This is achieved by releasing active compounds precisely at the intended site of action [52,53]. Nanoparticles must maintain high purity levels and exhibit uniformity in size and shape to predict their behavior accurately in diverse biological environments and minimize the likelihood of provoking an immune response, thus making them suitable for medical applications [54,55].

### PNPs in Tumor Therapy

Hydrophobic anticancer drugs administered intravenously through solvent-based delivery systems are commonly used in the treatment of various solid tumors. While these agents have shown significant clinical progress, their efficacy is often hindered by the toxicities associated with their solvent-based formulations [56,57]. Moreover, these formulations can impede the tumor penetration due to the formation of large polar micelles, resulting in nonlinear pharmacokinetics and a reduced unbound drug fraction. To address these obstacles, the utilization of various drug delivery systems has become prevalent in cancer therapy, leading to the creation of innovative and encouraging formulations [58]. Nano-drug delivery systems (NDDSs) offer a hopeful avenue for enhancing anticancer therapy, with the potential to significantly improve the in vivo distribution, circulation time, behavior, tissue specificity, cellular uptake, and overall performance of therapeutic agents [59,60,61]. Proteins have been used as the ideal material for nanoparticle preparation and drug delivery due to their unique characteristics and amphiphilicity, enabling interactions with both the drug and the solvent. Thus, PNPs have been employed as nanocarriers for anticancer medications to enhance their antitumor efficacy [62,63,64]. In addition, proteins can be derived from a variety of sources, further enhancing their versatility. Natural proteins, such as water-soluble proteins like bovine and human serum albumin, as well as insoluble proteins like the zein water-soluble proline-rich protein fraction from corn, and gliadin, can be employed in synthesizing these nanoparticles [65].

Albumin, due to its high number of negative charges and inherent ability to transport hydrophobic molecules, has been extensively employed as a nanocarrier for anticancer agents [66]. In a study, the authors developed an albumin nanoparticle conjugated to doxorubicin and a monoclonal antibody [67]. The produced nanoparticles exhibited precise targeting towards melanoma cells. Additionally, nanoparticles carrying doxorubicin demonstrated enhanced cytotoxic effects on melanoma cells compared to the unbound drug [67]. Another study incorporated succinylated cholesterol into bovine serum albumin (BSA) and proceeded with stirring under an argon atmosphere [68]. The dissolution of cholesteryl (Chol)–BSA was followed by the gradual addition of paclitaxel to create an albumin–drug composite (paclitaxel–Chol–BSA) under stirring conditions. The resulting paclitaxel–Chol–BSA structure exhibited superior colloidal stability. These paclitaxel–Chol–BSA nanoparticles functioned effectively as they displayed sustained drug release. The administration of paclitaxel–Chol–BSA nanoparticles led to an increased cellular uptake in comparison to paclitaxel alone. Furthermore, the assessment of the cytotoxicity of paclitaxel–Chol–BSA nanoparticles against cancerous cells revealed a reduced viability and heightened cytotoxicity induced by paclitaxel when delivered through these nanoparticles, as opposed to paclitaxel alone used as a control [68]. A study in phase I was conducted to analyze the toxicity profile and pharmacokinetics of the albumin/paclitaxel complex. Nineteen patients diagnosed with advanced solid tumors were administered the complex. There were no instances of acute hypersensitivity reactions or unforeseen toxicities, and the hematologic toxicity was reported to be mild and non-cumulative [69]. Subsequent research indicated that the drug clearance and volume of distribution were notably higher for the albumin/paclitaxel complex [70]. In a phase II trial conducted across multiple centers, 63 female participants with metastatic breast cancer were administered albumin-bound paclitaxel through intravenous infusion [71]. The overall response rate observed in all patients was 48%. Specifically, patients who received nab-paclitaxel as either the first-line or subsequent therapy for their metastatic disease exhibited response rates of 64% and 21%, respectively. The median time to disease progression was recorded at 26.6 weeks, while the median survival duration was 63.6 weeks. Notably, no severe hypersensitivity reactions were documented during the phase II study, despite the absence of premedication. Furthermore, the toxicities associated with this treatment were significantly lower compared to those reported for high doses of paclitaxel administered as a standalone agent [71]. In a phase III trial involving 460 women with metastatic breast cancer, it was observed that the albumin/paclitaxel complex led to increased response rates in comparison to the paclitaxel alone, as well as an extended time to tumor progression [72].

Ferritin, a protein with a cage-like structure that is present in nearly all living organisms, can disassemble reversibly at either an extremely acidic pH of 2.5 or a basic pH of 13.0. Subsequently, the protein can undergo self-assembly to form a properly folded protein nanocage at a neutral pH [73]. Due to these unique nanocage properties, ferritins have been effectively utilized for drug loading purposes. It has been documented that ferritin can selectively target tumor cells that exhibit an overexpression of the transferrin receptor, TfR1 (CD71). Human ferritin heavy chain (HFt) has been employed as a carrier for efficiently delivering chemotherapeutic agents like doxorubicin to cancer cells [74].

Gelatin, renowned for its biocompatibility, biodegradability, affordability, and abundance of active groups for attaching targeting molecules, stands as a highly adaptable natural biopolymer extensively employed in the pharmaceutical sector [75]. The utilization of gelatin nanoparticles (GPs) as carriers for cisplatin (CDDP) was employed in the development of a polymer–anticancer drug conjugate. The results from the study indicated that the nanoparticle/drug complex exhibited a higher potency compared to free CDDP. This heightened effectiveness was attributed to the complex’s rapid impact on the cell cycle and its superior inhibition of cell growth. In addition, the GP/CDDP complex exhibited stronger anti-tumor activity and reduced toxicity in comparison to free CDDP. The targeting ability, anticancer effect, and delivery of these nanoparticle/drug complexes were confirmed through both in vitro and in vivo experiments. Furthermore, the successful demonstration of aerosol delivery for the nanodrug carrier emphasized the potential utility of simple aerosol delivery in the clinical treatment of lung cancer patients [76]. Table 1 shows a summary of the information of the commonly used PNPs for cancer therapy.

Table 1 showcases the presence of encouraging data stemming from in vitro and in vivo studies, supporting the potential application of these PNPs in cancer therapy. However, it is important to note that clinical trials have predominantly concentrated on albumin-bound drugs. Hence, further extensive research appears necessary for exploring the potential of other PNPs in this context.

To facilitate the production of PNPs for drug delivery, various techniques can be employed. Each technique yields distinct outcomes in terms of nanoparticle size, morphology, and other properties. In order to develop effective PNPs for tumor therapy, it is necessary to understand the nanoparticle production methods, so that the nanoparticle properties can be optimized to enhance drug delivery and minimize the off-target effects, ultimately resulting in a positive outcome for cancer patients.

## 3. Methods for Generating PNPs

The convergence of nanotechnology and biotechnology has sparked innovative approaches to fabricate PNPs, offering versatile applications across numerous domains [37]. In contemporary research, PNPs derived from viruses, virus-like particles, ferritins, and enzyme complexes, have gained attention for their diverse utilities in catalysis, materials engineering, drug delivery, gene therapy, vaccine design, and biomedical imaging [78]. Numerous techniques are available for producing PNPs, with the selection of a specific method depending on factors such as the physico-chemical properties of the protein, its amino acid composition, and the characteristics of the intended drug payload [26,79]. Generally, two main approaches are employed: bottom-up and top-down. Top-down methods involve physically reducing bulk particles into smaller sizes, offering versatility across diverse fields like medicine and materials science for tailored nanomaterial fabrication. Bottom-up approaches, on the other hand, utilize fundamental atoms and molecules to construct nanoparticles, typically through chemical means [80].

As the generated particles in a top-down approach are typically of a significant size, and the technique is time consuming, bottom-up techniques are the primary methods employed for the synthesis of PNPs [81]. The bottom-up process encompasses various techniques such as desolvation, coacervation, salting out, nanoprecipitation, emulsification, nano spray drying, self-assembly, microfluidic technique, and nanoparticle albumin-bound (NAB) technology [82].

**Figure 2:**
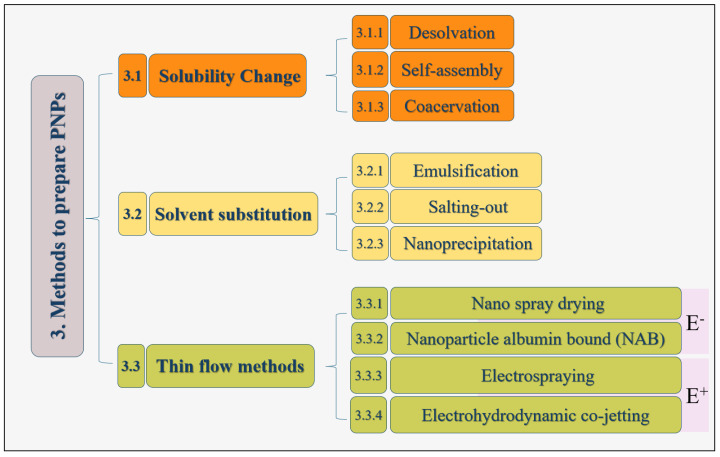
Overview of various techniques utilized to produce PNPs, illustrating diverse methods and their respective processes. E^+^ stands for the presence of electric field whereas E^−^ stands for the absence of electric field.

Regarding PNP synthesis, various techniques can be classified based on the shared procedural steps. Here, these techniques are categorized into three main groups: solubility change, solvent substitution, and thin flow methods, illustrated in Figure 2. Alongside the existing techniques, advanced methods have also driven the field of nanomedicine forward by offering precise control over particle characteristics and capabilities. Regardless of the technique, nanoparticle generation involves unfolding proteins and reducing the hydrophobic intramolecular interactions within the protein. The conformational changes during nanoparticle formation are influenced by factors like protein composition, concentration, the preparation conditions (e.g., pH, ionic strength, solvent), and crosslinking techniques [83,84].

### 3.1. Solubility Change

In chemistry, solubility refers to how well a solute mixes with a solvent. Desolvation, self-assembly, and coacervation are three methods within this category. They adjust the solubility of the target protein to induce nanoparticle formation during synthesis.

#### 3.1.1. Desolvation

**Figure 3:**
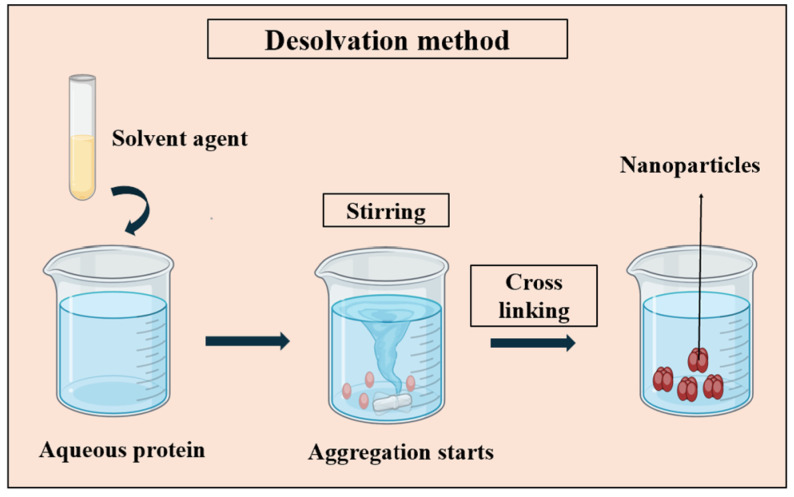
Illustration of the desolvation method employed in PNP synthesis, demonstrating the process of solvent removal to induce protein aggregation and PNPs’ formation using crosslinking agents such as glutaraldehyde.

In general, the desolvation process involves the introduction of a desolvating agent, such as alcohol or acetone, into a protein solution in water while stirring. This results in the dehydration of the protein and a change in its conformation to a coiled state. To increase the density of the nanoparticles and protect the coacervates, the amino functional groups of the protein can be crosslinked by crosslinking agents such as glutaraldehyde. This is the most commonly used crosslinker and changes the structure of protein by bridging the free amino groups of lysine or hydroxylysine residues [26]. Figure 3 represents the schematic concept of the desolvation method.

In 1978, Marty et al. [85] pioneered the use of a straightforward desolvation technique for producing gelatin nanoparticles. Building on this, Coester et al. [86] introduced a crucial modification, transitioning to a two-step process to address the particle aggregation issues. By employing ethanol as the desolvating agent and introducing a re-desolvation step, they managed to produce stable nanoparticles with a diameter of 277 nanometers. In 2003, Langer et al. [87] developed a pump-regulated technique, incorporating a tubing pump to generate human serum albumin (HSA) nanoparticles. Their findings underscored the significant influence of pH on particle size, with smaller particles observed at higher pH levels. Esfahlan et al. in 2016 [88], successfully utilized a specially designed apparatus to produce bovine serum albumin (BSA) and HSA nanoparticles, showcasing the advancements in the synthesis methods. More recently, Sozer et al. [89] implemented a cationization step alongside salicylic acid addition to enhance the drug loading capacity in BSA nanoparticles. Additionally, Khramtsov et al. [90] introduced a rapid one-step desolvation technique for gelatin nanoparticle synthesis, demonstrating the favorable colloidal stability and non-toxicity across the various gelatin types. These developments collectively contribute to the ongoing refinement and versatility of this nanoparticle synthesis method.

In some instances, nanoparticles prepared using this method may still lack long-term stability and can quickly undergo redissolution. Thus, a second desolvation step becomes necessary to achieve smaller and more uniform nanoparticles. Despite its widespread use due to its simplicity, scalability, and cost-effectiveness, the desolvation technique has two notable drawbacks: reliance on organic solvents and the use of toxic crosslinkers [26,91,92].

#### 3.1.2. Self-Assembly

**Figure 4:**
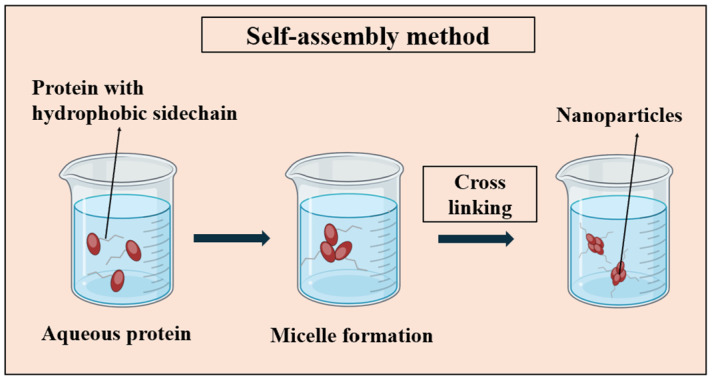
Illustration depicting the self-assembly method utilized for PNP synthesis, highlighting the spontaneous organization of protein molecules into nanoparticles through non-covalent interactions such as hydrogen bonding, hydrophobic interactions, and electrostatic forces. The crosslinking step is performed using crosslinking agents such as glutaraldehyde.

The initial methods of self-assembly heavily relied on utilizing the self-assembling patterns observed in nature. In the natural world, various proteins demonstrate spontaneous bonding, forming PNP complexes, including viruses, ferritin, and encapsulin. These nanoparticles consist of natural proteins mingling via inherent protein–protein interactions [93]. Leveraging this potential, natural, modified, or de novo protein micelles can spontaneously form when individual protein chains are dissolved in an aqueous solution. These micellar structures can be further reinforced and stabilized through crosslinking (i.e., glutaraldehyde) among the polymer chains. These crosslinked micelles, at the nanoscale, exhibit robust stability even upon dilution and remain resilient to changes in the experimental conditions such as pH, ionic strength, solvents, and shear forces [26]. Figure 4 illustrates the basic steps involved in the self-assembly process.

In 2007, Semo et al. [94] utilized the self-assembly properties of casein molecules to produce nano-capsules, incorporating vitamin D2 into the process to evaluate the encapsulation mechanisms and micelle protection. This approach significantly improved the stability and bioavailability of the hydrophobic bioactive compound, curcumin, through encapsulation [95]. Gong et al. [96] modified albumin and employed self-assembly for paclitaxel (PTX) incorporation, achieving notable drug-loading and entrapment efficiency, with PTX-loaded micelles ranging from 123.3 to 152.8 nm in size. Elzoghby et al. [97] integrated spray drying into casein-derived micellar nano-vehicles to enhance the bacter-tumor efficacy of flutamide, resulting in nanoparticles sized between 63 and 93 nm and improved anti-tumor effects. Yuan et al. [98] combined partial enzymatic hydrolysis and self-assembly techniques to create soy PNPs for curcumin delivery, yielding nanoparticles with a diameter of 80 nm. In 2022, Pakdel et al. [55] successfully generated exceedingly small keratin nanoparticles (22.83 nm) that exhibited a homogeneous size and shape. This accomplishment was achieved by harnessing the inherent self-assembling ability of keratin molecules to synthesize nanostructures.

In contrast to the alternative methods, protein self-assembly offers the formation of well-defined and highly conserved clusters. Although specifying individual interactions is challenging due to their complexity, these interactions demonstrate site-specificity and orientation dependence, resulting in an orderly and uniform nanoparticle distribution [22]. The resulting PNP conjugates exhibit a high degree of conservation, displaying specific behavior at distinct sites and maintaining order and uniformity. However, this system’s success relies heavily on the precise arrangement at the atomic level. Immunogenicity is a concern due to mutated or newly designed constituent proteins. Moreover, the stability of self-assembled PNPs in the bloodstream is critical, considering the potential disruptions due to the complex intermolecular interactions. Despite these challenges, the exceptional molecular precision and diverse protein design opportunities make this approach promising for nanoparticle-based delivery systems [79].

#### 3.1.3. Coacervation

**Figure 5:**
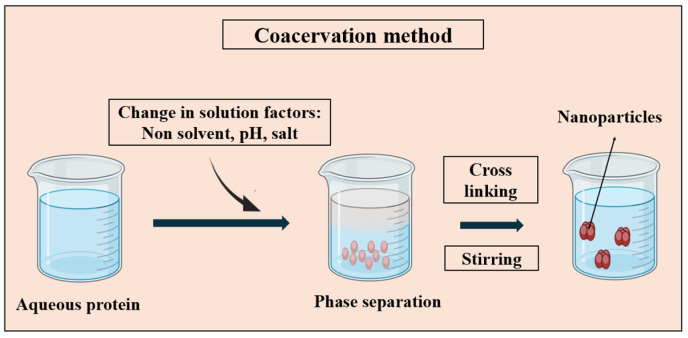
Schematic representation of the coacervation method for PNP synthesis, depicting the phase separation process resulting in the formation of PNPs using crosslinking agents such as glutaraldehyde.

The coacervation process relies on the modulation of protein solubility in solvents, affected by factors like solvent polarity, pH, ionic strength, and electrolyte presence [26,99]. Illustrated in Figure 5, this method induces phase separation by reducing the protein solubility. The introduction of salt into the protein solution triggers liquid–liquid phase separation, yielding a dense polymer-rich phase at the bottom and a clear solution above. This separation facilitates the creation of nanoparticles with the desired properties.

Oppenheim and Stewart pioneered the utilization of the coacervation phase separation technique in 1979 [100]. Through the addition of salts, they were able to create gelatin and albumin nanoparticles measuring around 500 nm in size. In 1997, Leo and colleagues [101] introduced to the procedure a crosslinking stage by using glutaraldehyde and incorporated doxorubicin during the crosslinking phase. The nanoparticles obtained exhibited a size range of 100–200 nm. In 2010, HSA nanoparticles were synthesized by Sebak et al. [102] through the pH-coacervation technique, resulting in drug-loaded nanoparticles ranging in size from 150 to 300 nm. Meiguni and colleagues [103] have recently employed a complex coacervation technique in the production of bean protein and Arabic gum nanoparticles, aimed at encapsulating curcumin. The resulting particles demonstrated a size distribution spanning from 168 to 4027 nm.

Coacervation stands out for its simplicity, cost-effectiveness, scalability, and reliable encapsulation performance. However, its susceptibility to pH and ionic strength presents a notable challenge for commercial use. Coacervation typically occurs within a narrow pH range, often below the protein’s isoelectric point, complicating the encapsulation of pH-sensitive materials. Moreover, even minor salt concentrations can destabilize complex coacervates, demanding salt-free conditions [104].

### 3.2. Solvent Substitution

The methods in this category involve a change in the solvent used in the protein solution, inducing conformational changes in protein particles, and yielding nanoparticles. Three techniques classified under this group are emulsification, salting out, and nanoprecipitation.

#### 3.2.1. Emulsification Method

**Figure 6:**
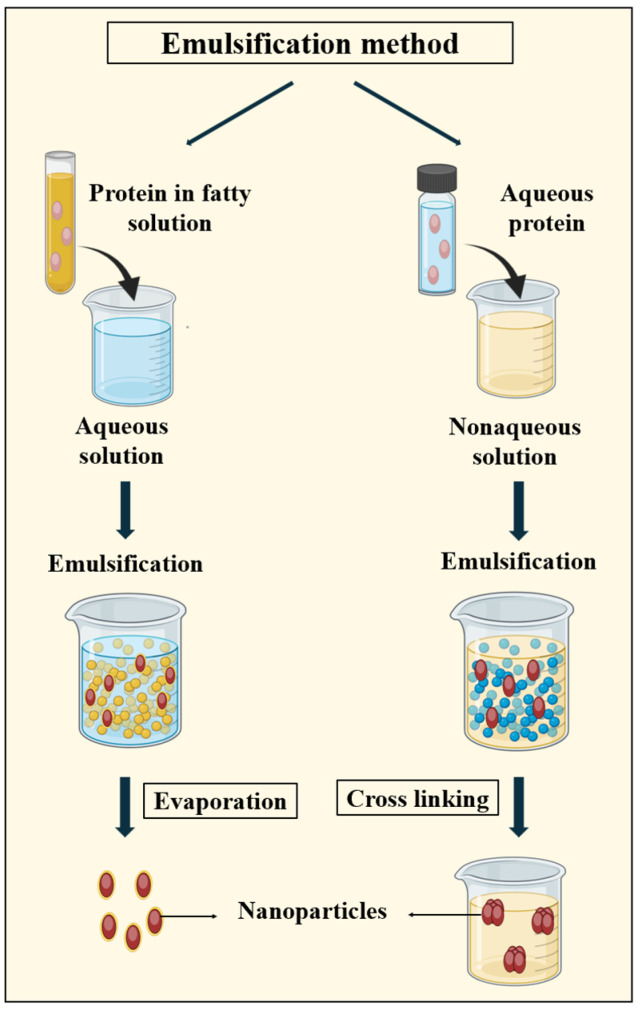
Illustration of the emulsification method used in PNP synthesis, demonstrating the process of protein dispersion within an oil or water phase to form emulsion droplets, followed by stabilization to produce PNPs. The crosslinking step is performed using crosslinking agents such as glutaraldehyde.

Emulsions consist of mixtures of two liquid phases that are unable to mix but can be combined through the application of mechanical shear, typically stabilized by surfactants to prevent coalescence. The classification of emulsions varies based on factors such as the composition of the continuous phase, the order in which the components are added, and the type of surfactants used. The most basic types of emulsions are single emulsions, including water-in-oil (W/O) and oil-in-water (O/W) emulsions. As shown in Figure 6, by acting as a barrier between the two phases, surfactants enable the formation of relatively stable nanoparticles within the emulsion. Surfactants play a crucial role in the production of PNPs by minimizing the surface tension between the aqueous protein solution and the oily phase [105,106].

Following the PNP formation by using this method, external crosslinking is necessary, typically achieved through chemical crosslinkers or heat denaturation methods [79,105,106]. In 1995, Leroux et al. [107] introduced the emulsification method, laying the groundwork for the subsequent research. They produced gelatin nanoparticles averaging 700 nm in size. In 2002, Cascone et al. [108] developed methotrexate-loaded gelatin nanoparticles (100–200 nm) via a one-step water-in-oil emulsification method crosslinked with glutaraldehyde. Elzoghby et al. [109] innovatively incorporated an ionic crosslinking process into an oil-in-water emulsification technique, resulting in casein nanoparticles under 100 nm in size, enhancing the circulation time and blood clearance of flutamide. Later, Girgis et al. [110] utilized a double emulsion technique, known as water-in-oil-in-water (W/O/W), to produce BSA nanoparticles ranging from 295 to 408 nm in size.

However, the advancements in PNP production via emulsification have been limited, attributed to various factors. Firstly, proteins’ amphiphilic nature makes them better emulsifiers than final product components. Additionally, the resulting particle sizes (~430 nm) and loading efficiency (~2.21%) are suboptimal. Despite these challenges, emulsification remains notable for its convenience and minimal equipment requirements, making it accessible for laboratory use [111].

#### 3.2.2. Salting out

**Figure 7:**
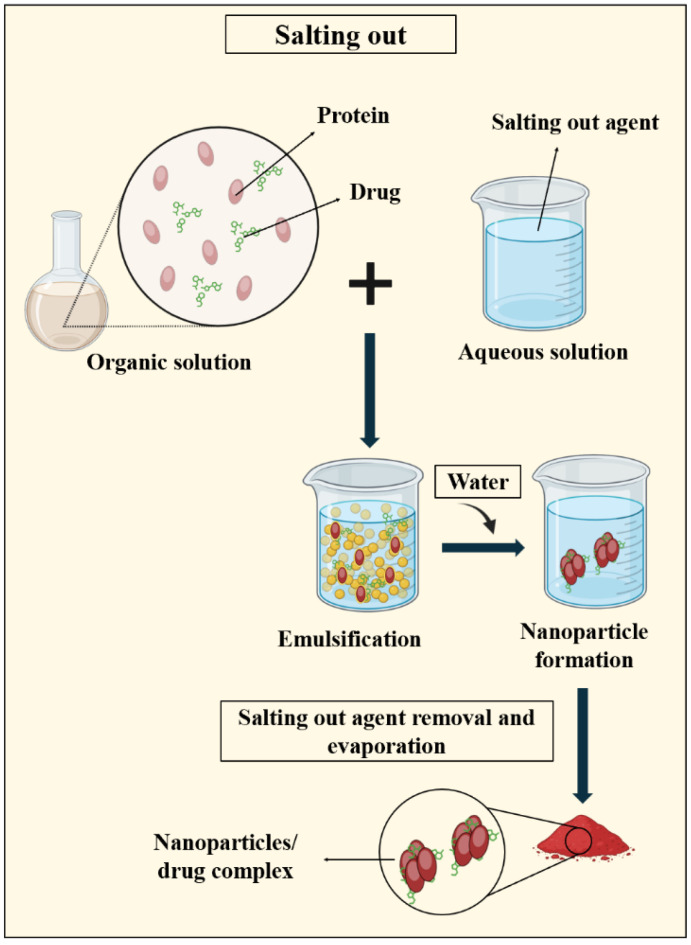
Schematic representation of the salting out method employed in PNP synthesis, demonstrating the precipitation of proteins from solution by the addition of a high concentration of salts, leading to the formation of PNPs.

The salting out principle involves separating a water-miscible solvent from an aqueous solution via the salting out effect. In general, both the protein and drug are dissolved in a solvent like acetone, which is then emulsified in an aqueous gel containing a salting out agent and a colloidal stabilizer. Dilution with water or an aqueous solution facilitates acetone diffusion into the aqueous phase, leading to nanosphere formation. Solvent and salting out agent removal is accomplished through cross flow filtration (Figure 7) [26,112].

The utilization of the salting out technique has a historical origin in the production of polymeric nano-dispersions, which leads to the formation of nanoparticles within the low micrometer range of 427-938 nm [113]. In 2010, Lammel and colleagues [114] employed the salting out method to produce silk fibroin nanoparticles utilizing potassium phosphate salt. At the minimal protein concentration, nanoparticles ranging in size from 489 nm to 1.2 µm were successfully generated.

The salting out technique offers several benefits, including the reduction in stress on protein carriers. Unlike other methods, this technique does not require any temperature adjustments, making it ideal for heat-sensitive substances. However, there are limitations to this approach, specifically its applicability only to lipophilic drugs and the expensive washing step required for nanoparticles [26,112].

#### 3.2.3. Nanoprecipitation

**Figure 8:**
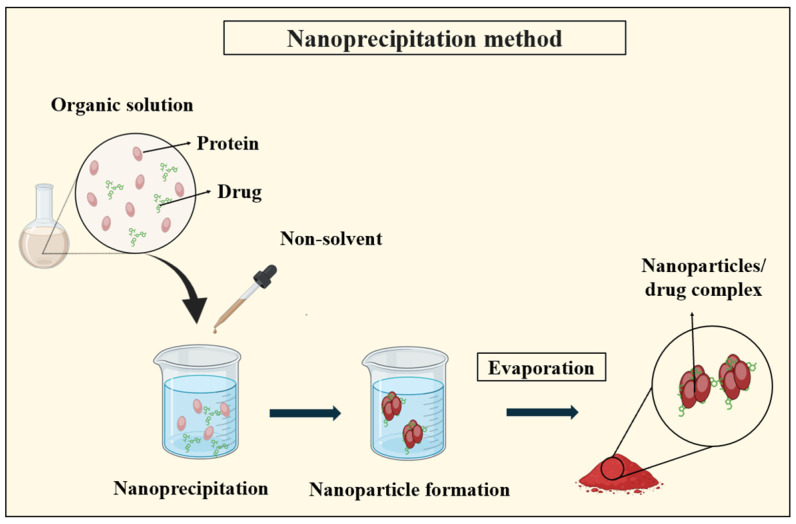
Illustration depicting the nanoprecipitation method for PNP synthesis, demonstrating the rapid mixing of a protein solution with a non-solvent to induce nanoparticle formation through precipitation.

The nanoprecipitation or solvent displacement technique relies on the utilization of two miscible solvents, where both the protein and the drug must be soluble in one solvent while remaining insoluble in the other. This process initiates when the protein solution encounters the non-solvent, triggering rapid desolvation and nanoprecipitation. As depicted in Figure 8, as the protein-containing solvent diffuses into the dispersing medium, polymer precipitation occurs, entrapping the drug [115].

Patel et al. [116] utilized nanoprecipitation to synthesize zein nanoparticles loaded with curcumin, resulting in a mean particle size ranging from 100 to 150 nm. Lee et al. [117] improved the stability by incorporating an emulsifier into the process, yielding gelatin nanoparticles sized between 200 and 500 nm. Khan and Shneider [118] compared nanoprecipitation with the desolvation method, noting the differences in particle size distribution, with nanoprecipitation resulting in 210 nm particles and desolvation producing 60 nm particles. Zhao et al. [119] innovatively utilized flash nanoprecipitation, combining nanoprecipitation with regulated nucleation and growth to fabricate controlled-size PNPs.

Another variation involves adding a protein solution in 70–80% aqueous alcohol containing the drug to water with a stabilizer under vigorous stirring. As the alcohol concentration decreases, the protein precipitates, forming nanoparticles. This method is suitable for hydrophobic proteins like zein and gliadin, offering the rapid and easy fabrication of small-sized particles with a unimodal distribution. Compared to the traditional emulsification techniques, nanoprecipitation avoids the need for intense shear forces to decrease the droplet sizes [26,111].

### 3.3. Thin Flow Methods

Thin flow methods typically involve a liquid confined between a solid substrate and a free surface in contact with another fluid, such as air [120]. Within this domain, two distinct sub-categories have emerged, with one incorporating electric fields and the other not. The former includes methods like electro spraying and electrohydrodynamic co-jetting, which utilize electric potential differences of several kilovolts (5-34 KV) [121], while the latter encompasses techniques such as nano spray drying and NAB technology, which do not involve electric potential.

#### 3.3.1. Nano Spray Drying

**Figure 9:**
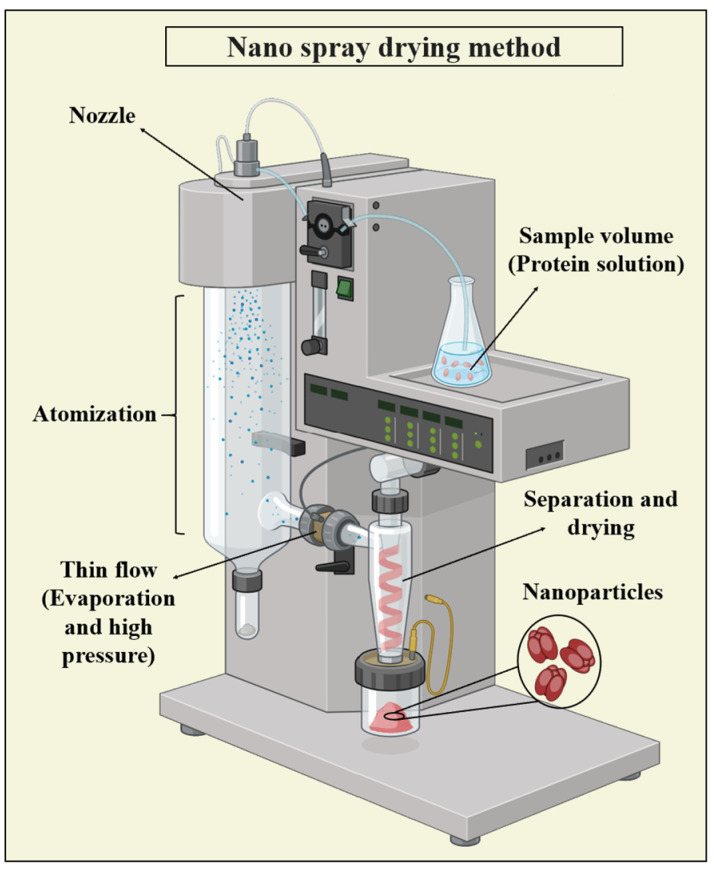
Schematic representation of the nano spray drying method used for PNP synthesis, illustrating the process of atomization of protein solution into fine droplets followed by rapid drying to form PNPs.

Spray drying is a method used to convert liquids into particles continuously, offering protection to delicate molecules like proteins from degradation. It allows for the precise control over particle properties such as the size, bulk density, and flow characteristics by adjusting various parameters. This technique is extensively employed in commercial protein drug production. The process involves four stages: (1) converting the nanoparticle suspension into a fine spray, (2) interaction between the spray and surrounding air, (3) moisture evaporation from the spray, and (4) isolating the final dried product from the drying gas [79,111,122]. Figure 9 illustrates the general concept of nano spray drying.

Nano spray drying, widely used across industries, offers efficiency and hands-off operation, producing solid PNPs from an initial aqueous formulation input. The continuous improvements in spray dryer design have led to smaller, more uniform particles and an increased throughput. While this method shows promise, its performance compared to other techniques remains unclear due to the limited studies and ongoing instrument advancements. Success hinges on its ability to produce particles with competitive characteristics, particularly their size, which is crucial for nanomedicine fabrication [123].

#### 3.3.2. Nanoparticle Albumin-Bound (NAB) Technology

**Figure 10:**
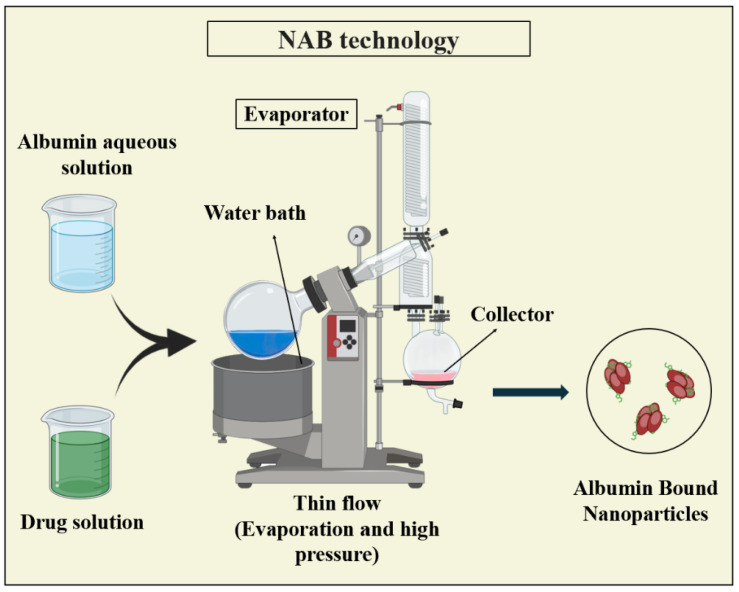
Illustration depicting the NAB technology used for PNP synthesis, highlighting the encapsulation of proteins within albumin-based nanoparticles for enhanced stability and targeted delivery.

NAB technology is widely used for encapsulating poorly water-soluble drugs, providing a safe intravenous administration method. In NAB, the drug and albumin, mostly human serum albumin, are combined in a water-based solvent and subjected to high pressure through a jet, resulting in drug–albumin nanoparticles typically sized between 100 and 200 nm [79,124,125]. Figure 10 illustrates NAB technology.

NAB technology offers advantages such as delivering hydrophobic drugs without using harmful solubilizers. Unlike traditional emulsification, NAB does not require external crosslinking for stability, eliminates the need for surfactants, and offers greater process control [126]. However, limitations may arise from the availability of suitable stabilizing agents lacking natural sulfhydryl and/or disulfide crosslinking groups. Nonetheless, NAB technology has made significant strides in various applications [79].

This method was utilized in the production of NAB-paclitaxel, which is the first nanotechnology-based drug approved by the FDA and known by the commercial name, Abraxane. Initially developed for treating metastatic breast cancer, it has now been used for the treatment of various cancer types, including pancreatic cancer [64,77].

#### 3.3.3. Electrospraying

**Figure 11:**
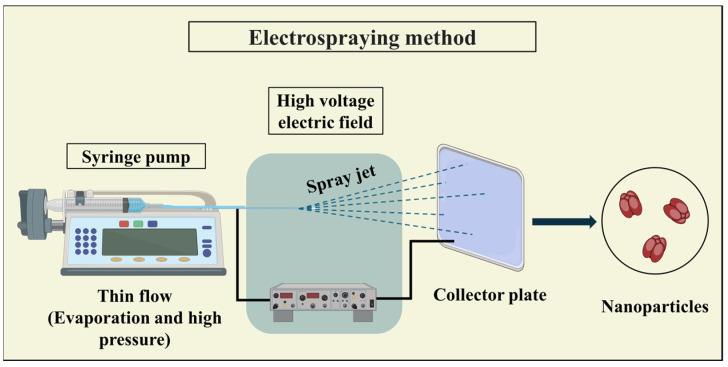
Schematic representation of the electrospraying method utilized in PNP synthesis, illustrating the process of electrostatically charging protein droplets and directing them towards a collector, where they form nanoparticles upon solvent evaporation.

Protein-based particles, including fibers and capsules, can be synthesized using electrospray techniques. This involves applying an electric field to draw a protein solution through a small nozzle. Initially, a mixture of protein and drug is dispersed in a suitable solvent and placed in a capillary tube. The protein solution is then extracted from the tube by subjecting it to a high-voltage electric field, forming a thin jet. The resulting particle type, whether fibers or capsules, depends on the electric field strength and protein solution characteristics. During the electrospray process, solvent evaporation occurs as the jet travels from the capillary to the collector (Figure 11). To ensure complete solvent evaporation, a sufficient distance between the capillary and collector is essential to prevent particle fusion [79,111,127].

Electrospraying offers advantages over conventional methods as it does not require templates or surfactants to create nanoparticles and provides high drug loading efficiency and self-dispersion capabilities. However, a limitation of electrospraying arises from the inability to use certain proteins independently due to their complex macromolecular and 3-dimensional structures, along with strong inter- and intramolecular forces. To overcome this limitation, surfactants, plasticizers, or reducing agents can be incorporated into the protein solutions [26,111].

#### 3.3.4. Electrohydrodynamic (EHD) Co-Jetting

**Figure 12:**
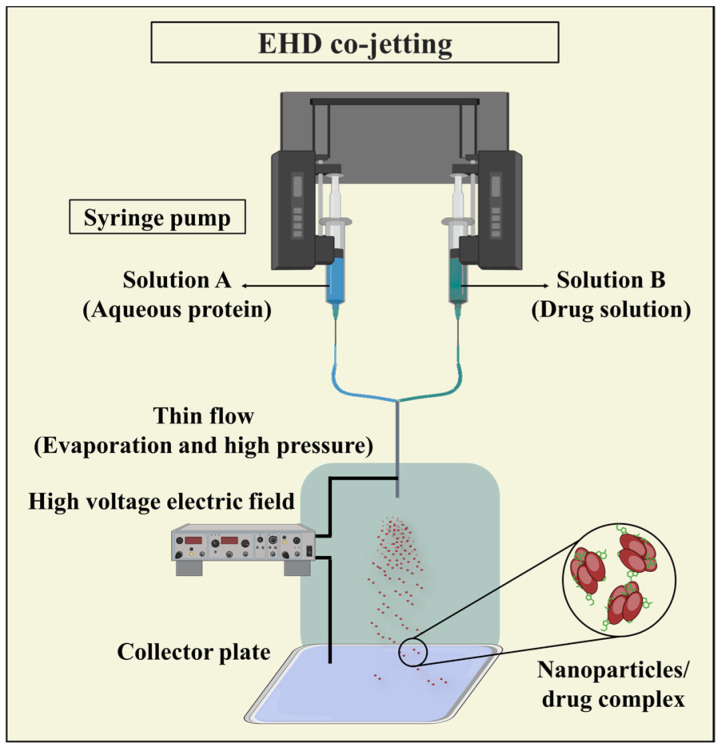
Illustration depicting the EHD co-jetting method employed in PNP synthesis, showcasing the simultaneous jetting of protein and drug solutions through electrically charged capillaries to form composite nanoparticles with controlled properties.

EHD co-jetting has emerged as a valuable technique for crafting intricate particles, including multicompartmental micro- and nanoparticles, with broad applications in drug delivery. In EHD co-jetting, multiple needles arranged side by side serve as capillaries, receiving input solutions at controlled flow rates to ensure laminar flow and a stable interface between jetting solutions. As droplets form at the needle tips, applying an electric field causes the meniscus to distort. This forms a Taylor cone, from which an electrified jet is generated. This jet breaks up into charged droplets that undergo rapid solvent evaporation and solidification of nonvolatile components (Figure 12). The swift solvent evaporation preserves the initial geometry of the input solutions, enabling the fabrication of intricate particle architectures that would otherwise be challenging to achieve [128,129].

EHD co-jetting offers several advantages, including the preservation of protein biological activity and structure during fabrication, adjustable process parameters, the ability to incorporate various therapeutic agents, and the generation of compartmentalized nanoparticles with enhanced tunability. However, a drawback of electrospraying is its limited throughput, which ongoing efforts aim to address through the implementation of a needleless electrospraying technique [79].

**Figure 13:**
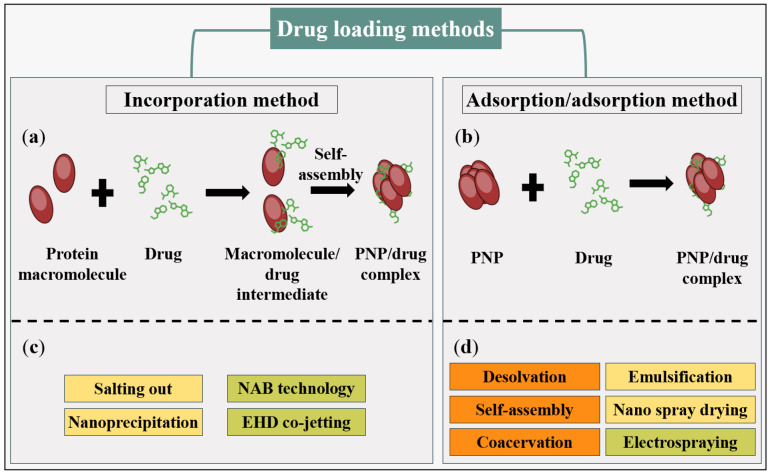
Schematic diagram of drug loading methods, namely (**a**) incorporation method; (**b**) adsorption/adsorption method; (**c**,**d**) their related PNP preparation methods, each utilizing a distinct drug loading technique to obtain the final PNP/drug complex.

It should be noted that drug loading into the nanoparticle system can be achieved through two distinct methods: the first method involves incorporating the drug during the preparation of nanoparticles and producing an intermediate complex, known as the incorporation method. The second method entails mixing the carrier with a concentrated drug solution after the nanoparticles have been prepared, referred to as the adsorption/adsorption technique [130]. In the second method, mechanisms like adsorption, electrostatic interactions, entrapment, and hydrophobic forces are used. Figure 13 illustrates these drug loading methods.

## 4. Comparing PNP and PNP/Drug Complex Synthesis Methods

A detailed examination of the various methods for synthesizing PNPs is presented in Table 2. This includes the information on the size of the nanoparticles produced by each method, along with the respective advantages and disadvantages associated with these techniques. Controlling the size of PNPs is essential because it directly influences their behavior and performance in both medical and industrial contexts. For instance, in medical application and drug delivery, the size of the nanoparticles can affect their biodistribution, cellular uptake, and therapeutic efficacy. Similarly, in industrial settings, the size of PNPs can impact their stability, functionality, and suitability for specific manufacturing processes.

Like any process, each method used in PNP synthesis comes with its own set of benefits and drawbacks, making them appropriate for different industrial applications. For example, the solubility change methods, such as desolvation and coacervation, rely on altering the solubility of proteins to induce nanoparticle formation. These methods are advantageous due to their simplicity and scalability, making them suitable for large-scale industrial production. However, they often require the use of organic solvents and may involve complex purification processes, limiting their applicability for certain sensitive drugs or biological materials. On the other hand, the solvent substitution techniques, including emulsification and nanoprecipitation, offer versatility and control over nanoparticle size and properties. Furthermore, they are advantageous for encapsulating hydrophobic drugs and can be tailored to suit the various protein types and drug formulations. Nevertheless, the solvent substitution methods may require the optimization of the process parameters and can be sensitive to the changes in the solvent composition, affecting the reproducibility and scalability in industrial settings. In contrast, the thin flow methods, such as electrospraying and electrohydrodynamic co-jetting, enable the fabrication of intricate nanoparticle structures with precise control over the morphology and composition. Moreover, these methods are particularly advantageous for producing complex multicompartmental nanoparticles and are suitable for applications requiring precise drug delivery mechanisms. However, the thin flow methods often involve specialized equipment and may have lower throughput compared to other techniques, limiting their industrial scalability. As a result, while the solubility change methods offer simplicity and scalability, the solvent substitution techniques provide versatility and control, and the thin flow methods enable the precise fabrication of complex nanoparticle structures. Each method presents unique advantages and challenges, and their suitability for industrial applications depends on factors such as the specific drug formulation, desired nanoparticle properties, and available manufacturing infrastructure.

## 5. Advanced Technologies for PNP Synthesis

The advanced methods for the production of PNPs have significantly developed the field of nanomedicine, offering the precise control over particle properties and functionality. One such method is pulse power technology, which utilizes pulsed electric fields to efficiently synthesize PNPs, offering several advantages such as the precise control over the particle size, morphology, and surface properties [49]. These nanoparticles find applications in various industries, including drug delivery, biomedical imaging, tissue engineering, and diagnostics.

**Figure 14:**
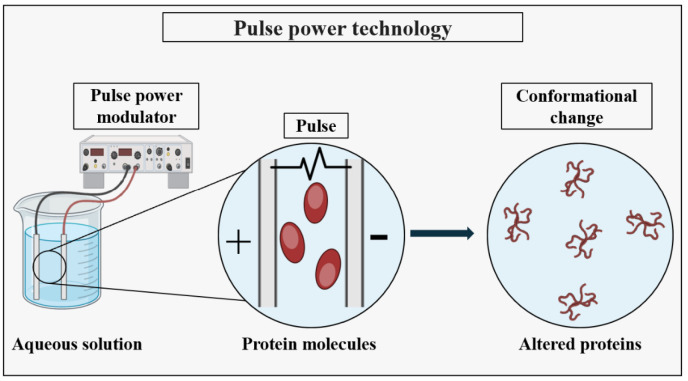
The schematic representation illustrates the impact of pulse power on the structure of cellular proteins, demonstrating the alteration in protein conformation following the application of pulse power.

Pulsed power technology has emerged as a significant approach in the synthesis of metal nanoparticles (MNPs). Its simplicity, rapidity, and eco-friendly nature have attracted increasing attention in recent times [131]. This technology facilitates the production of nanoparticles with customized properties, which are highly desirable for drug delivery purposes, including sustained release and targeted delivery [132]. Although there is currently no existing literature on the utilization of pulse power technology for the production of PNPs, it has been demonstrated that this technology can induce conformational changes in proteins by directly or indirectly influencing their secondary and tertiary structures as shown in Figure 14 [133,134,135]. As highlighted earlier in this article, conformational alterations play a crucial role in the formation of PNPs. Consequently, it is reasonable to anticipate that the pulse power-assisted synthesis of PNPs could be feasible. By employing pulse power-assisted synthesis, PNPs can be created with precise control over their porosity and size, rendering them suitable for a wide range of applications.

Microfluidics-based techniques provide another avenue for the precise manipulation of fluid flows at the microscale, enabling the fabrication of PNPs with controlled composition and structure [48]. Furthermore, the advanced biomanufacturing methods, such as recombinant DNA technology and cell-free protein synthesis, offer the ability to produce PNPs with customizable functionalities tailored for specific biomedical applications [11,37]. These advanced approaches hold immense promise for the development of the next-generation therapeutics, diagnostics, and biomedical devices, facilitating the advancements in personalized and precision medicine [26].

## 6. Conclusions

PNPs represent a remarkable advancement in tumor therapy, offering a myriad of benefits and customizable properties that make them an attractive choice for targeted drug delivery. Their biocompatibility and low toxicity ensure minimal harm to healthy tissues, while their high specificity enables the precise targeting of tumor sites, maximizing the treatment efficacy while minimizing the adverse effects. Moreover, their biodegradability facilitates the safe clearance from the body, reducing the risk of long-term complications. The versatility of proteins allows for the tailored customization of nanoparticle properties, enhancing the drug loading capacity and control over the release kinetics, thus optimizing the therapeutic outcomes. Additionally, the ability to incorporate various therapeutic payloads further enhances their potential for comprehensive tumor eradication. Despite the metabolic and conformational changes experienced by PNPs upon exposure to a biological setting, their efficacy as a nanocarrier has been validated in clinical studies, indicating that these modifications have been considered. Moreover, the clearance rate of PNPs has been demonstrated to prevent alterations due to oxidative stress, affirming their safety. However, further investigation is necessary to expand our knowledge of these modifications in nanoparticles in drug delivery.

This review offers a comprehensive examination of the various methods for PNP production, delving into the challenges and considerations inherent in each approach. The present study classified PNP synthesis methods into three main categories based on their common procedure steps—the solubility change, solvent substitution, and thin flow methods. The categorization system presented here holds several advantages. One of its primary benefits is that it presents a clear and straightforward framework for organizing a wide range of techniques used in nanoparticle synthesis, which helps to simplify the understanding of the underlying mechanisms. Additionally, these categories have a direct correlation with the behavior of proteins in diverse settings, underscoring the significance of solubility, interactions with solvents, and fluid dynamics in nanoparticle formation. Furthermore, these categories can be applied to a variety of proteins and biomolecules, providing flexibility and adaptability for the different experimental conditions and application requirements. Finally, the clarity of this categorization system facilitates the easy comparison and evaluation of the different synthesis methods within each group, aiding in the identification of the best practices, emerging trends, and areas for further research.

The latest progress in PNP synthesis has been centered on enhancing the efficiency, versatility, and specificity of the techniques employed in nanoparticle production. A notable advancement has been the development of innovative protein engineering strategies aimed at tailoring the structure and properties of the protein carriers for drug delivery. Furthermore, researchers have explored new fabrication techniques, such as 3D bioprinting and microfluidics, for precisely controlling the size, shape, and composition of nanoparticles. In addition, there has been significant progress in the utilization of natural biomaterials and environmentally friendly processes for nanoparticle synthesis, aiming to improve the biocompatibility and sustainability. Lastly, the integration of the advanced characterization techniques such as cryogenic electron microscopy and mass spectrometry (backscattered electron (BSE) imaging, nano-secondary ion mass spectrometry (SIMS)) has enabled the detailed analysis of PNP structures and functionalities, facilitating the design of the next-generation drug delivery systems with enhanced efficacy and safety profiles. These recent strides underscore the dynamic nature of PNP production and its potential to advance healthcare and biotechnology.

## Figures and Tables

**Table 1:** A summary of commonly used PNPs in cancer therapy.

PNPs Name	Drug Name	Targeted Cancer	Clinical Trials	References
Albumin (HSA and BSA)	Doxorubicin Paclitaxel	Breast cancer Melanoma cells Pancreatic cancer	Phase I Phase II Phase III	[67,68,69,70,71,72,77]
Ferritin	Doxorubicin	Most cancer cells with high expression of the transferrin receptor TfR1	N/A	[73,74]
Gelatin	Cisplatin	Lung cancer	N/A	[75,76]

**Table 2:** A comparative overview of the techniques delineating the size spectrum of generated PNPs, merits, and demerits within each category of methodologies.

Method	PNPs Size (nm)	Advantages	Disadvantages	References
Solubility change	24–1000<	Simplicity Cost effective Scalability	Low PNPs stability Toxicity Immunogenicity	[26,78,85,86,87,88,89,90,92,93,96,98,99,100,101,102]
Solvent substitution	49–900<	Feasibility Cost effective	Low loading efficiency Immunogenicity Some limitations on drug variety	[26,105,106,107,108,109,110,111,112,114,115,116,117]
Thin flow methods	87–1000<	Hands-off operation Final solid PNPs High PNPs stability High loading efficiency	Limited studies Instrument advancement High cost Some limitations on protein variety	[26,38,78,109,120,122,124,126,127]

## Data Availability

Data are contained within the article.
